# The tree shrew is a promising model for the study of influenza B virus infection

**DOI:** 10.1186/s12985-019-1171-3

**Published:** 2019-06-07

**Authors:** Bing Yuan, Chunguang Yang, Xueshan Xia, Mark Zanin, Sook-san Wong, Fan Yang, Jixiang Chang, Zhitong Mai, Jin Zhao, Yunhui Zhang, Runfeng Li, Nanshan Zhong, Zifeng Yang

**Affiliations:** 1 Department of Respiration, the First People’s Hospital of Yunnan Province, Kunming, Yunnan 650032 People’s Republic of China; 2 The Affiliated Hospital of Kunming University of Science and Technology, Kunming, Yunnan 650032 People’s Republic of China; 3 State Key Laboratory of Respiratory Disease, National Clinical Research Center for Respiratory Disease, Guangzhou Institute of Respiratory Health, the First Affiliated Hospital of Guangzhou Medical University, Guangzhou, Guangdong 510120 People’s Republic of China; 4 Faculty of Life Science and Technology, Kunming University of Science And Technology, Kunming, Yunnan 650500 People’s Republic of China; 5 Medical Faculty, Kunming University of Science And Technology, Kunming, Yunnan 650500 People’s Republic of China; 6 State Key Laboratory of Quality Research in Chinese Medicine, Macau University of Science and Technology, Avenida Wai Long, Taipa, Macau, People’s Republic of China

**Keywords:** Influenza B virus, Tree shrew, Ferret, Mouse, Animal model, List of Abbreviations.

## Abstract

**Background:**

Influenza B virus is a main causative pathogen of annual influenza epidemics, however, research on influenza B virus in general lags behind that on influenza A viruses, one of the important reasons is studies on influenza B viruses in animal models are limited. Here we investigated the tree shrew as a potential model for influenza B virus studies.

**Methods:**

Tree shrews and ferrets were inoculated with either a Yamagata or Victoria lineage influenza B virus. Symptoms including nasal discharge and weight loss were observed. Nasal wash and respiratory tissues were collected at 2, 4 and 6 days post inoculation (DPI). Viral titers were measured in nasal washes and tissues were used for pathological examination and extraction of mRNA for measurement of cytokine expression.

**Results:**

Clinical signs and pathological changes were also evident in the respiratory tracts of tree shrews and ferrets. Although nasal symptoms including sneezing and rhinorrhea were evident in ferrets infected with influenza B virus, tree shrews showed no significant respiratory symptoms, only milder nasal secretions appeared. Weight loss was observed in tree shrews but not ferrets. V0215 and Y12 replicated in all three animal (ferrets, tree shrews and mice) models with peak titers evident on 2DPI. There were no significant differences in peak viral titers in ferrets and tree shrews inoculated with Y12 at 2 and 4DPI, but viral titers were detected at 6DPI in tree shrews. Tree shrews infected with influenza B virus showed similar seroconversion and respiratory tract pathology to ferrets. Elevated levels of cytokines were detected in the tissues isolated from the respiratory tract after infection with either V0215 or Y12 compared to the levels in the uninfected control in both animals. Overall, the tree shrew was sensitive to infection and disease by influenza B virus.

**Conclusion:**

The tree shrew to be a promising model for influenza B virus research.

**Electronic supplementary material:**

The online version of this article (10.1186/s12985-019-1171-3) contains supplementary material, which is available to authorized users.

## Highlights

- Ferrets, tree shrews and mice were infected by influenza B virus strains of Yamagata or Victoria lineage isolated during our clinical surveillance. Viruses caused 100% mortality in BALB/c mice.
- Viral replication and clinical signs of influenza were similar between tree shrews and ferrets, although weight loss was only observed in tree shrews. These data show that tree shrews are a useful alternative to ferrets for studies of influenza B viruses, particularly considering their relatively small size, evolutionary relatedness to primates and lower cost compared to ferrets.

## Background

Influenza B viruses (IBVs) are one of the main causes of seasonal influenza cases, accounting for up to 40% of total cases [1]. However, during the 2011/2012 and 2015/2016 influenza seasons in China, 53.7 and 52.2% of influenza notifications were due to IBV infections [2]. There are two lineages of IBVs currently in circulation; B/Victoria/2/1987-like (Victoria) and B/Yamagata/16/1988-like (Yamagata). Generally, IBVs cause a less severe respiratory disease compared to influenza A viruses (IAVs) [3]. However, IBVs cause more complications in children, including neurological, muscular [4] and cardiac-associated events [5]. Recent studies also showed that the antiviral drug Oseltamivir, the drug most commonly used to treat influenza, was less effective against IBV compared to IAV [6–8].

The tree shrew (*Tupaia belangeri*), is a small non-primate mammal that has been used as an animal model to study viral infection [9]. Tree shrews are a potentially promising model for the study of IBVs as they are more closely related to primates compared to ferrets, which are commonly used in influenza virus research [10, 11]. Tree shrews are also smaller than ferrets, easier to handle and more cost-effective. Unlike mice, tree shrews display symptoms including fever, rhinitis and mild pneumonia post inoculation with IAVs, and show a distribution of α2,3-linked and α2,6-linked sialic acids in their respiratory tract similar to humans [10, 12]. As such, here we conducted a study to determine if the tree shrew is a useful animal model for the study of IBV infection. We compared the tree shrew to two animal models commonly used in influenza research; ferrets and mice, using two strains of IBVs from Yamagata and Victoria lineages that were isolated from clinical specimens. Overall, we found the tree shrew to be sensitive to IBV inoculation, displaying a clear symptomology, viral shedding and seroconversion, thus demonstrating their utility as a model for future studies of IBVs.

## Materials and methods

### Cells and viruses

IBVs B/Guangzhou/0215/2012 (Victoria-like, V0215) and B/Guangzhou/12/2016 (Yamagata-like, Y12) were obtained from the State Key Laboratory of Respiratory Disease of Guangzhou Medical University. These viruses were isolated during our previous study of IBVs isolated from human cases (partial data was published in Chinese) [13]. Briefly, our study of 8 viruses in the mouse model revealed that only the V0215 and Y12 viruses were capable of replication in the mouse model without prior adaptation. Therefore, these viruses were selected for this present study. Madin–Darby canine kidney (MDCK) cells (American Type Culture Collection) were cultured in Dulbecco’s Modified Eagle’s Medium (Gibco) supplemented with 10% fetal bovine serum. All the viruses were cultured in MDCK cells cultures in DMEM without bovine serum and containing 1 μg/mL of L-(tosylamido-2-phenyl) ethyl chloromethyl ketone (TPCK) treated trypsin (Sigma). Virus titers were determined via the 50% tissue culture infective dose (TCID_50_) method using MDCK cells and the Reed–Muench method [14].

### Animals

Male tree shrews weighing 100 to 155 g and special-pathogen-free female six-week-old BALB/c mice were obtained from the Animal Experimental Centre of Kunming Medical University. Female ferrets weighing 600 to 700 g were obtained from Wuxi Coral Reef Biotechnology Co., Ltd. (Wuxi city, Jiangsu province, China). Ferrets and tree shrews were confirmed to be seronegative by virus neutralization assay to V0215 and Y12 viruses prior to commencement of experiments. All the animals were fed in IVC cages in a BSL-2 animal house at the Animal Experimental Centre of Kunming Medical University. Animals were acclimatized to their housing for 2 days prior to use in these studies.

Animals were divided into four groups; Group One comprised of ten tree shrews and ten ferrets. These animals were implanted with transponder microchips (IPTT-300, Bio Medic Data Systems Inc., Seaford, Delaware) to allow daily monitoring of body temperature for 5 days prior to intranasal inoculation with 1.0 × 10^6^ TCID_50_ of V0215 (five ferrets and five tree shrews) or Y12 (five ferrets and five tree shrews) in 250 μL of phosphate buffered saline, pH 7.4 (PBS). After inoculation, body weight and temperature were measured daily and nasal washes were collected using 1 mL of PBS at 2, 4 and 6 days post inoculation (DPI) to measure viral titers. Serum were collected 21 DPI to measure influenza-specific antibody titers. Seroconversion of the animals was tested by HI assays [15].

Group Two comprised of seven ferrets and 21 tree shrews. Three ferrets and nine tree shrews were inoculated with 1.0 × 10^6^ TCID_50_ of V0215 or Y12 and one ferret and three tree shrews were intranasally inoculated with equal PBS as negative controls. At 2, 4 and 6 DPI one ferret and three tree shrews inoculated with each virus were killed to collect respiratory tissues for pathology studies and real time PCR. The animals inoculated with PBS were killed at 6 DPI.

Group Three consisted of 15 mice. Five mice were intranasally inoculated with either 1.0 × 10^6^TCID_50_ of V0215 or Y12 or equal PBS. These mice were observed for 15 days for mortality and morbidity.

Group four consisted of 35 mice. 15 mice were intranasally inoculated with 1.0 × 10^6^TCID_50_ of either virus in 50 μL of PBS and five were inoculated with equal PBS as a negative control. At 2, 4 and 6DPI five mice inoculated with either V0215 or Y12 virus were killed and lungs were collected for pathology studies and real time PCR. The five mice inoculated with PBS were killed at 6DPI and lungs were collected for pathology studies.

### Quantification of mRNA

Tissues were homogenized and total RNA was extracted using Trizol reagent (Invitrogen) according to manufacturer’s instructions. Reverse transcription was performed using PrimeScript RT Master Mix kit (TAKARA) according to manufacturer’s instructions. Real-time PCR was conducted using TB Green *premix Taq* II (TAKARA) to measure cytokine and chemokine mRNA. Glyceraldehyde-3-phosphate dehydrogenase (GAPDH) was used as an internal reference gene. The primers were designed with Primer Primier 5.0 (Additional file 1: Table S1).

### Histology

The tissues isolated from the respiratory tract were first fixed with 10% neutral buffered formalin, embedded in paraffin, sectioned at 3 μm and stained with hematoxylin and eosin (H&E) after dehydration, the pathological sections were observed and photographed under a microscope (Nikon, ECLIPSE Ni).

### Statistical analysis

Data were analyzed using SPSS (version 13.0). The data were expressed as mean ± standard deviation of the mean (SD). Significance was determined by single-tailed student’s t-test when comparing two groups, Multiple group comparisons were performed via one-way-ANOVA test base on variance homogeneity. *p* < 0.05 was considered significant.

## Results

### Influenza B viruses replicated in all animal models, causing lethal infections in mice

**Fig. 1:**
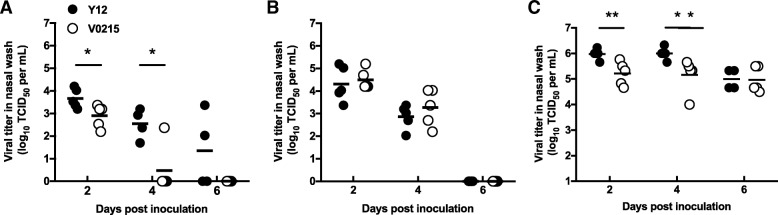
Influenza B viruses can replicate in tree shrews, ferrets and mice. Tree shrews (**a**), ferrets (**b**) and mice (**c**) were inoculated with 1.0 × 10^6^TCID_50_ of virus intranasally. At 2, 4 and 6 days post inoculation (DPI), nasal washes were collected and virus titers were determined by tissue culture infectious dose 50% (TCID_50_) assay in Madin Darby canine kidney (MDCK) cells●Yamagata strain B/Guangzhou/12/2016 (Y12), ○Victoria strain B/Guangzhou/0215/2012 (V0215). Lines indicate means. **p < 0.01, *p < 0.05

**Fig. 2:**
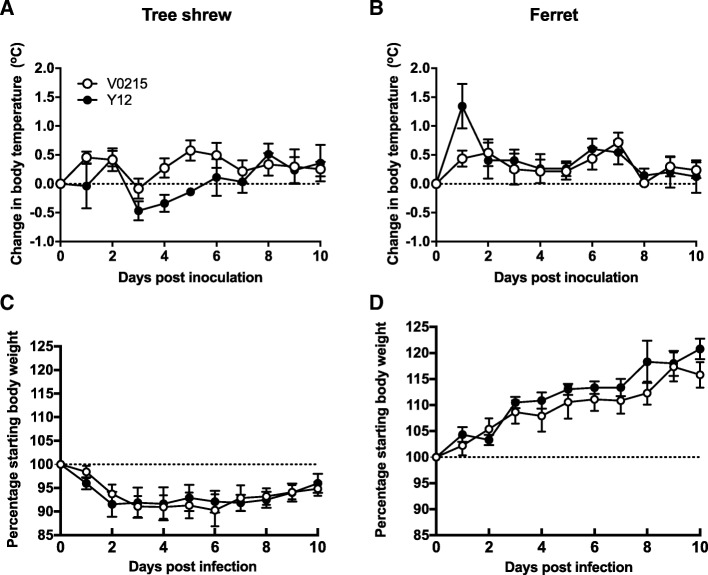
Tree shrews show weight loss post inoculation with influenza B viruses but ferrets do not. Tree shrews did not show an increase in body temperature greater than 0.5 °C above pre-inoculation (**a**) and up to 10% body weight loss (**c**) post inoculation with influenza B viruses. Ferrets inoculated with B/Guangzhou/12/2016 showed a mean body temperature increase of over 1 °C at one day post inoculation and at other timepoints temperatures were approximately 0.5 °C above pre-inoculation (**b**). Ferrets did not lose weight post inoculation with either virus (**d**). Body temperature and weight were measured daily after inoculation with 1.0 × 10^6^TCID_50_ of virus intranasally. The body temperature at 0 day was the average of five days before infection. ●Yamagata strain B/Guangzhou/12/2016 (Y12), ○Victoria strain B/Guangzhou/0215/2012 (V0215)

**Fig. 3:**
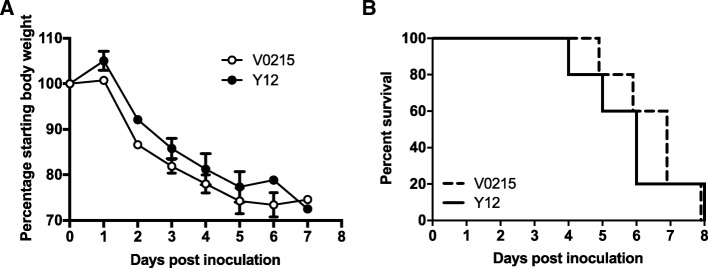
Both Yamagata and Victoria lineage influenza B viruses caused 100% mortality in BALB/c mice. Mice inoculated with either Yamagata strain B/Guangzhou/12/2016 (Y12) or Victoria strain B/Guangzhou/0215/2012 (V0215) began losing weight at two days post inoculation (**a**). All mice were killed by eight days post inoculation (**b**). ●Yamagata strain B/Guangzhou/12/2016 (Y12), ○Victoria strain B/Guangzhou/0215/2012 (V0215)

In our previous study we isolated IBVs from patients at the First Affiliated Hospital of Guangzhou Medical University [13]. As part of this study, we used 8 IBV strains to inoculate mice and we discovered two IBVs, V0215 and Y12, that caused severe disease without prior adaptation. In this current study we compared the susceptibility of tree shrews to ferrets and mice infected with V0215 and Y12 to determine the utility of tree shrews as models for future IBV studies. V0215 and Y12 replicated in all three animal models with peak viral titers evident on 2DPI (Fig. 1). There were no significant differences in titers between V0215 and Y12 inoculated ferrets, however, viral titers were significantly higher in tree shrews and mice inoculated with Y12 compared to V0215 at both 2 and 4DPI (Fig. 1a, b, c). There were no significant differences in peak viral titers in ferrets and tree shrews inoculated with Y12 at 2 and 4DPI, although viral titers were detected at 6DPI in tree shrews but not in ferrets. There was no mortality in tree shrews or ferrets and no significant differences in body temperature were observed in tree shrews or ferrets inoculated with either virus (Fig. 2a and b). Mean tree shrew body weight decreased post inoculation until 7DPI, with an overall mean weight loss of 10% (Fig. 2c). Weight loss was not detected in inoculated ferrets (Fig. 2d). Mice inoculated with either V0215 or Y12 began to lose weight at 2DPI and all mice were killed by 8DPI (Fig. 3). There were no significant differences in weight loss or mortality between mice inoculated with V0215 or Y12 (Fig. 3).

Seroconversion was observed in all tree shrews and ferrets. The antibody titer in ferrets and tree shrews inoculated with V0215 was significantly greater compared to Y12-inoculated animals (*p* < 0.05), indicating that the immunogenicity of V0215 was greater than Y12 (Table 1). Interestingly, HI titers measured in ferrets inoculated with either V0215 or Y12 were greater compared to tree shrews inoculated with those same viruses.

**Table 1:** Seroconversion and HI titers measured in tree shrews and ferrets 21 days post inoculation with influenza B viruses

Virus	Number of animals seroconverted (mean and range of HI titers)	
	Tree shrew	Ferret
V0215	5/5 (408, 40–640)	5/5 (1152, 640–2560)
Y12	4/4 (280, 160–320)	4/4 (560, 320–640)

### Expression of cytokine and chemokine mRNA

**Fig. 4:**
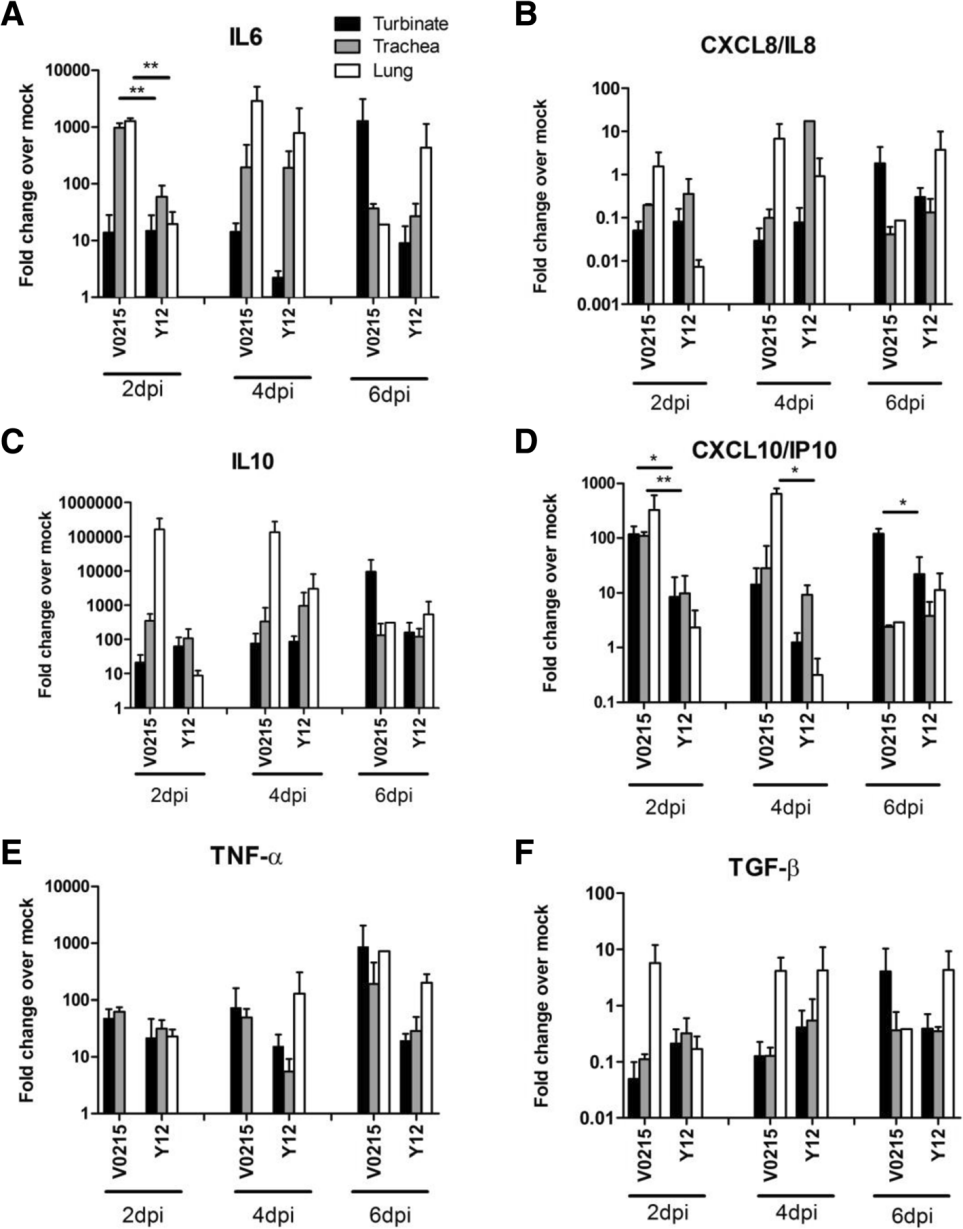
Inoculation of tree shrews with influenza B viruses causes inflammation in tissues of the respiratory tract. mRNA expression of IL-6 (**a**), IL-8 (**b**), IL-10 (**c**), IP-10 (**d**), TNF-α (**e**) and TGF-β (**f**) mRNA were increased at 2, 4 and 6 days post inoculation (DPI) in tissues of the respiratory tracts of tree shrews inoculated with influenza B virus. Statistically significant differences in fold expression levels of IL-6 (**a**) and IP-10 (**d**) were observed between tree shrews inoculated with the Yamagata or Victoria lineage virus. mRNA expression was detected via real-time PCR (SYBR Green) using GAPDH expression as a control. Fold changes are in comparison to uninoculated animals. Tree shrews were inoculated with either Yamagata strain B/Guangzhou/12/2016 (Y12) or Victoria strain B/Guangzhou/0215/2012 (V0215). **p < 0.01, *p < 0.05

**Fig. 5:**
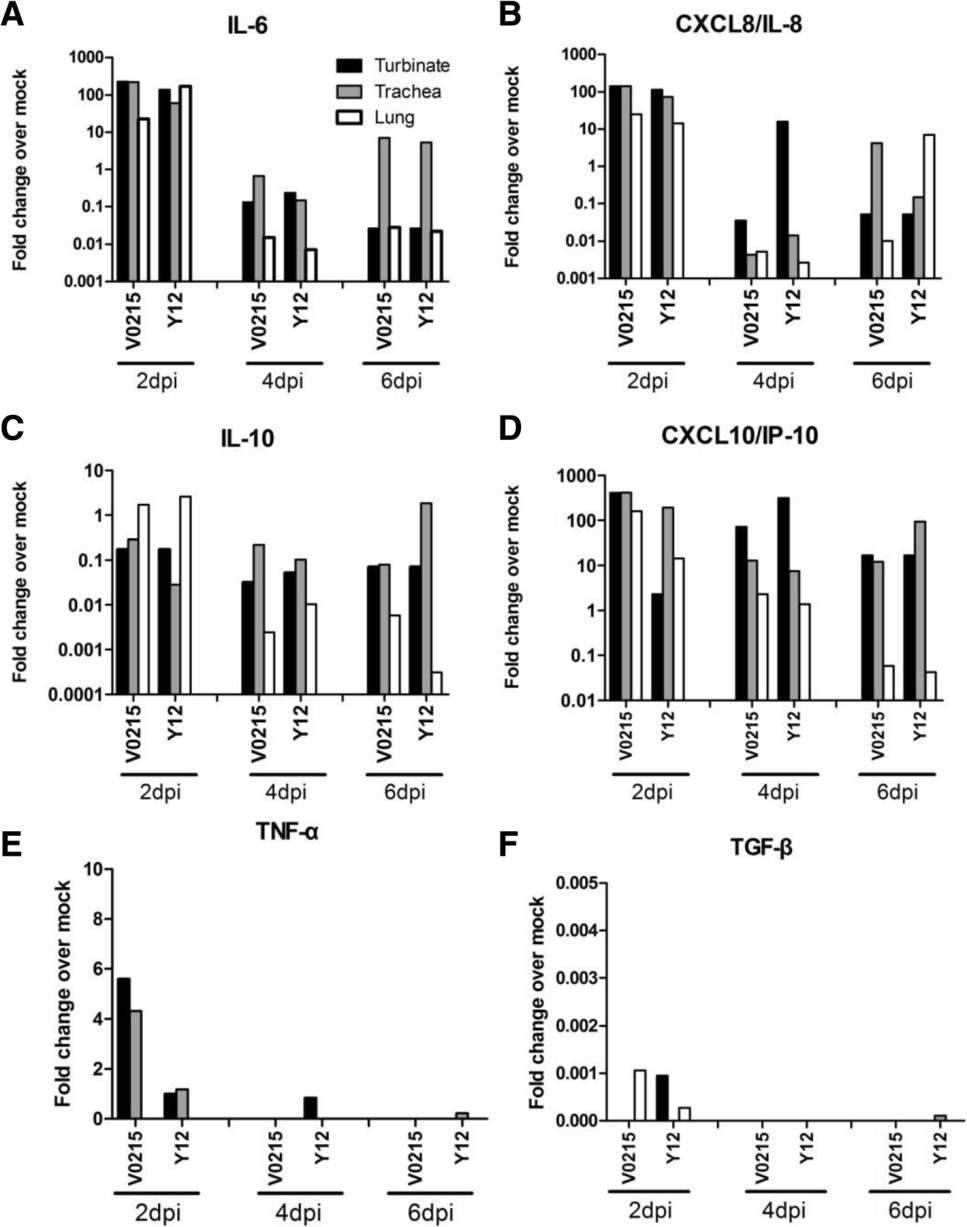
Inoculation of ferrets with influenza B viruses causes inflammation in tissues of the respiratory tract. mRNA expression of IL-6 (**a**), IL-8 (**b**), IL-10 (**c**), IP-10 (**d**), TNF-α (**e**) and TGF-β (**f**) mRNA were increased at 2, 4 and 6 days post inoculation (DPI) in tissues of the respiratory tracts of ferrets inoculated with influenza B virus. mRNA expression was detected via real-time PCR (SYBR Green) using GAPDH expression as a control. Fold changes are in comparison to uninoculated animals. Ferrets were inoculated with either Yamagata strain B/Guangzhou/12/2016 (Y12) or Victoria strain B/Guangzhou/0215/2012 (V0215)

**Fig. 6:**
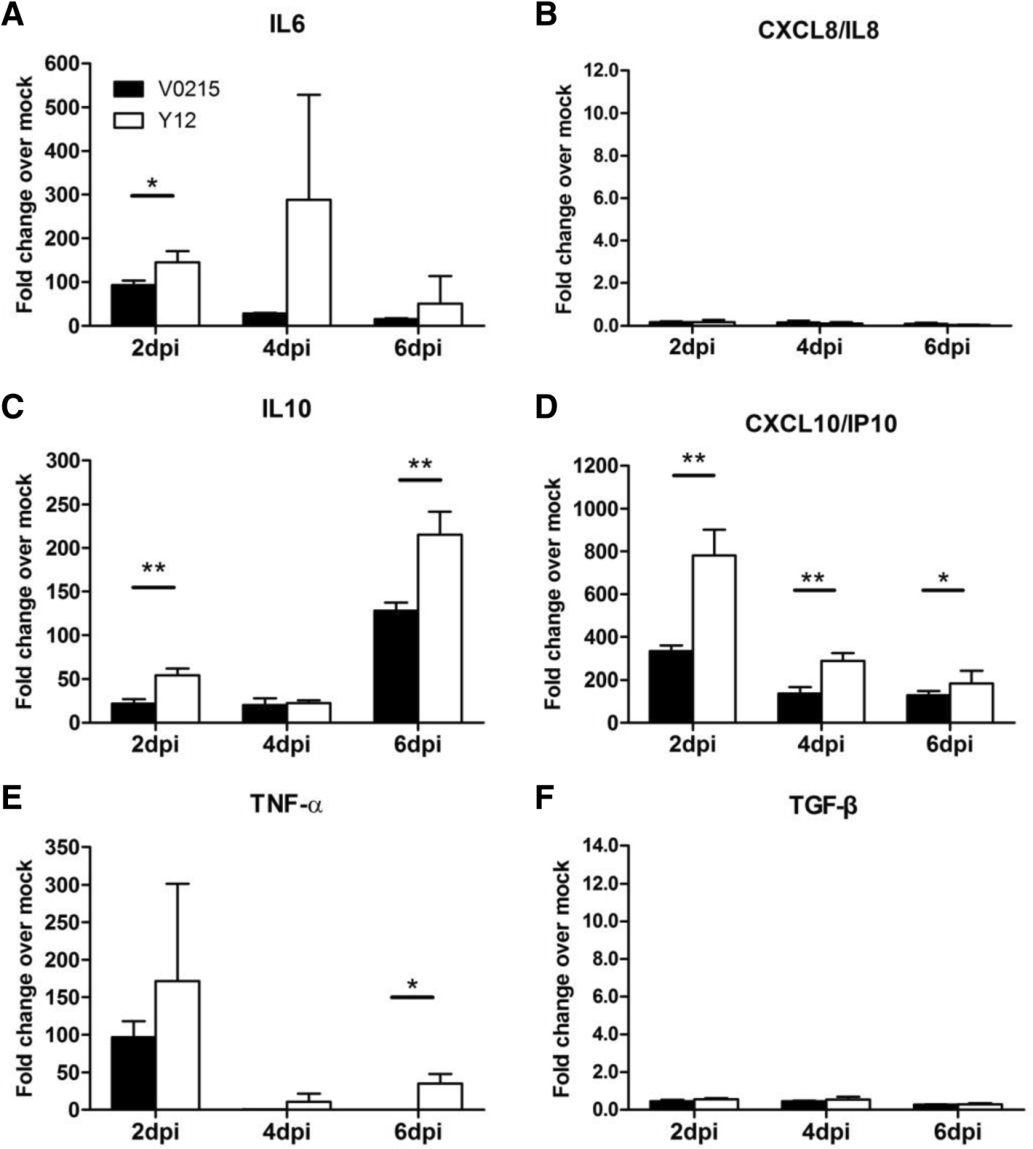
Inoculation of BALB/c mice with influenza B viruses causes inflammation in tissues of the respiratory tract. mRNA expression of IL-6 (**a**), IL-8 (**b**), IL-10 (**c**), IP-10 (**d**), TNF-α (**e**) and TGF-β (**f**) mRNA were increased at 2, 4 and 6 days post inoculation (DPI) in tissues of the respiratory tracts of mice inoculated with influenza B virus. The Yamagata strain B/Guangzhou/12/2016 elicited greater increases in the expression of IL-6 at two days post inoculation (DPI) (**a**), IL-10 at two and six DPI (**c**), IP-10 at all time points (**d**) and TNF-α at 6DPI (**e**). mRNA expression was detected via real-time PCR (SYBR Green) using GAPDH expression as a control. Fold changes are in comparison to uninoculated animals. Mice were inoculated with either Yamagata strain B/Guangzhou/12/2016 (Y12) or Victoria strain B/Guangzhou/0215/2012 (V0215)

Inflammation was observed in tissues of the respiratory tract obtained from tree shrews, ferrets and mice inoculated with either V0215 or Y12. Increased amounts of IL-6, IL-10, IP-10, TNF-α and TGF-β mRNA were detected at 2, 4 and 6DPI in tissues of the respiratory tract obtained from tree shrews (Fig. 4). V0215 elicited significantly greater expression of IL-6 and IP-10 mRNA at 2DPI compared to Y12 (Fig. 4a and d). In ferrets, amounts of IL-6, IL-8 and IP-10 mRNA were increased whilst IL-10 mRNA amounts were not appreciably increased post inoculation (Fig. 5). It should be noted that only one ferret per timepoint was used for cytokine analysis, meaning interpretation of this dataset is limited. In mouse lungs, increased amounts of IL-6, IL-10, IP-10 and TNF-α mRNA were detected at 2, 4 and 6DPI (Fig. 6). Y12 elicited a significantly greater expression of IL-6 at 2 and 4DPI, IL-10 at 2 and 6DPI, IP-10 at 2, 4 and 6 DPI and TNF-α at 6DPI compared to V0215 (Fig. 6).

### Pathological changes were observed in the respiratory tracts of tree shrews, ferrets and mice inoculated with V0215 of Y12

**Fig. 7:**
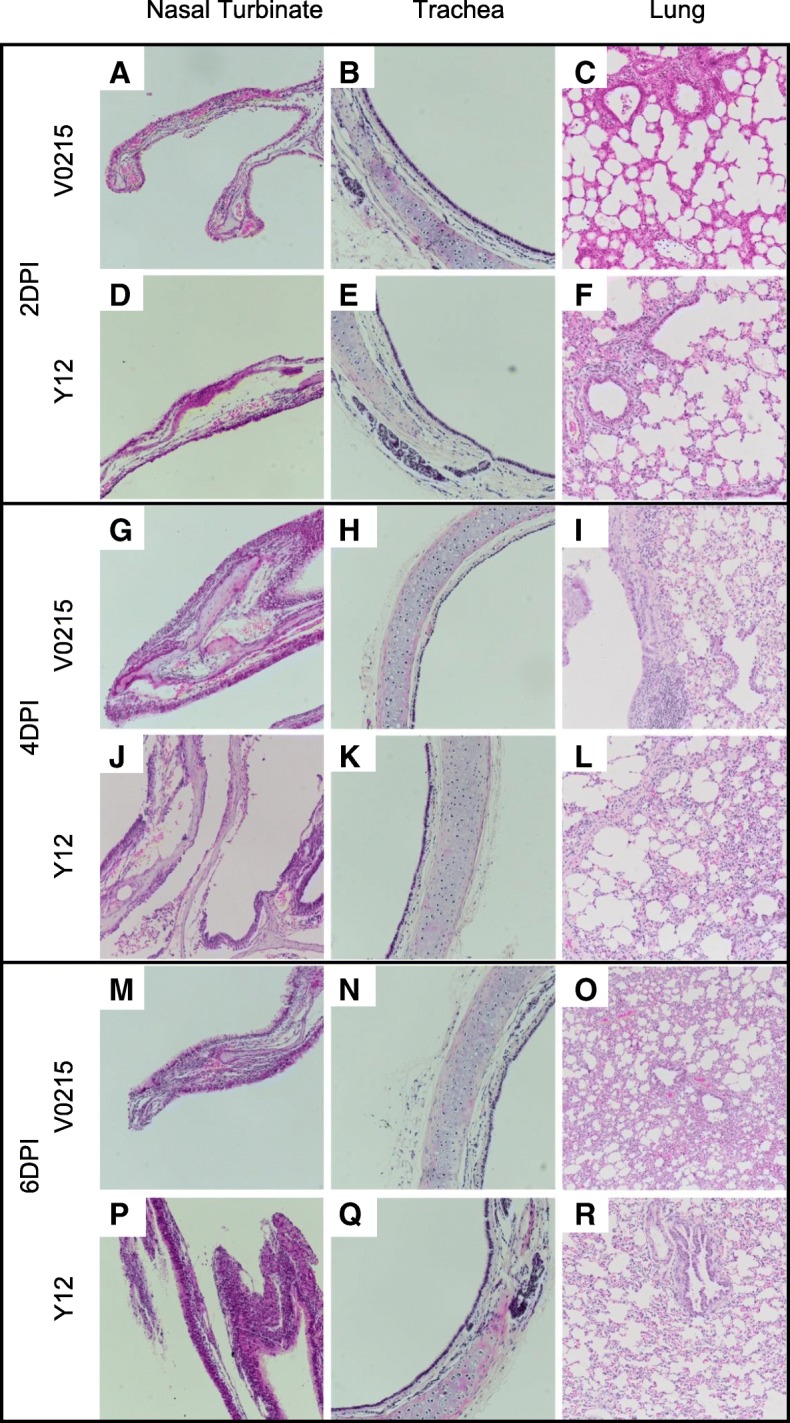
Influenza B virus inoculation caused pathology in the nasal turbinates, trachea and lungs of trees shrews. Nasal turbinates (**a**, **d**, **g**, **j**, **m**, **p**), trachea (**b**, **e**, **h**, **k**, **n**, **q**), and lungs (**c**, **f**, **i**, **l**, **o**, **r**) were collected at 2, 4 and 6 days post inoculation (DPI) from animals inoculated with either Yamagata strain B/Guangzhou/12/2016 or Victoria strain B/Guangzhou/0215/2012 and stained with hematoxylin and eosin. Images were examined at magnification of × 200

**Fig. 8:**
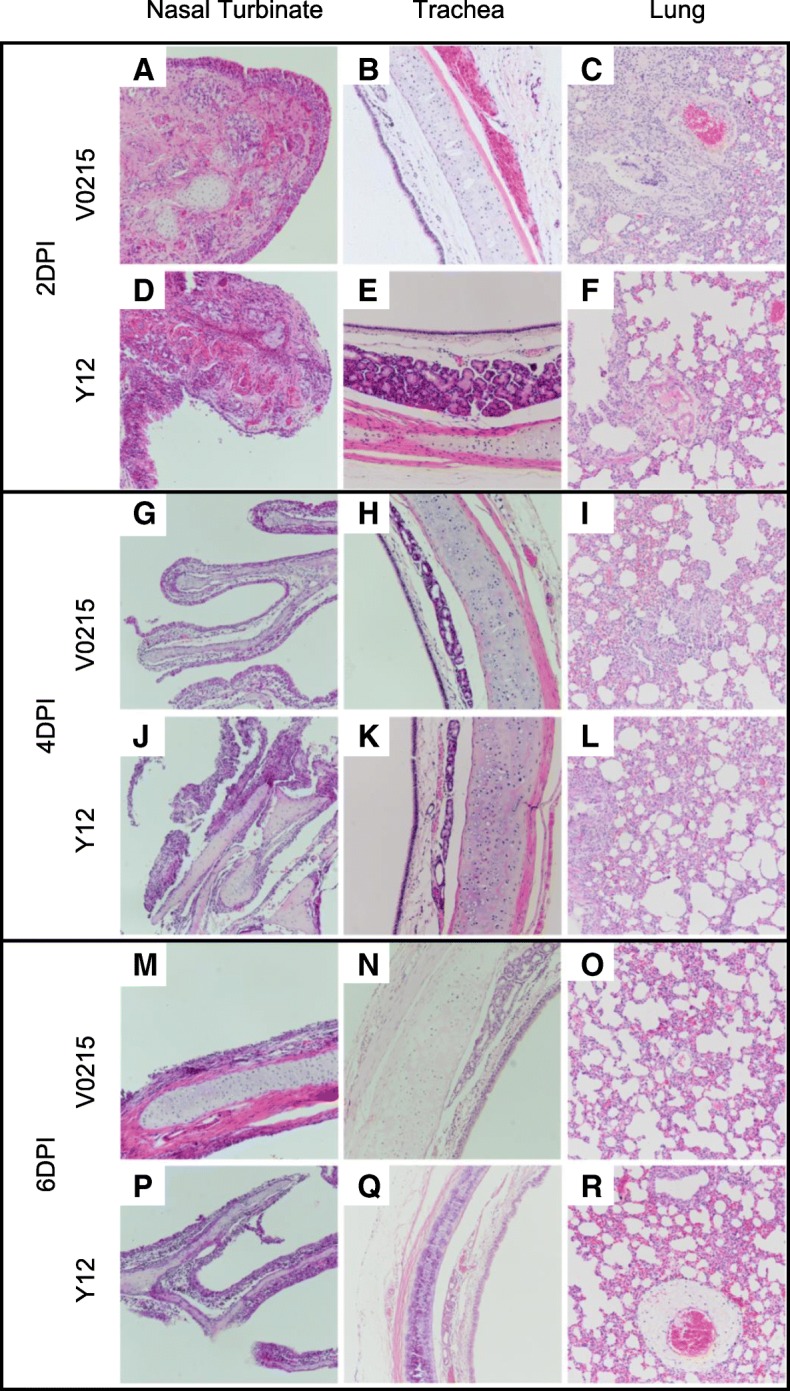
Influenza B virus inoculation caused pathology in the nasal turbinates, trachea and lungs of ferrets. Nasal turbinates (**a**, **d**, **g**, **j**, **m**, **p**), trachea (**b**, **e**, **h**, **k**, **n**, **q**), and lungs (**c**, **f**, **i**, **l**, **o**, **r**) were collected at 2, 4 and 6 days post inoculation (DPI) from animals inoculated with either Yamagata strain B/Guangzhou/12/2016 or Victoria strain B/Guangzhou/0215/2012 and stained with hematoxylin and eosin. Images were examined at magnification of × 200

**Fig. 9:**
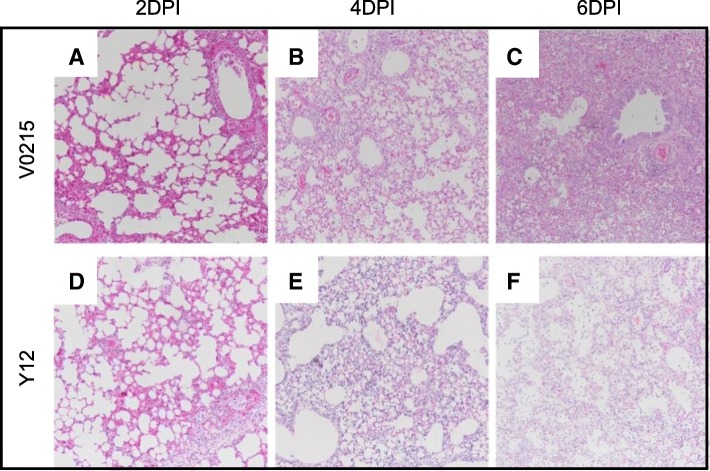
Influenza B virus inoculation caused pathology in the lungs of mice. Lungs were collected at 2 (**a**, **d**), 4 (**b**, **e**) and 6 (**c**, **f**) days post inoculation (DPI) from mice inoculated with either Yamagata strain B/Guangzhou/12/2016 or Victoria strain B/Guangzhou/0215/2012 and stained with hematoxylin and eosin. Images were examined at magnification of × 200

Histopathology of respiratory tissues obtained from tree shrews inoculated by IBVs revealed rhinitis and pneumonia in all animals, evident as submucosal hyperemia and focal edema and the presence of inflammatory cells (Figs. 7, 8 and 9). Evidence of mild rhinitis in six of nine tree shrews inoculated with V0215 was also evident (Fig. 7). Two tree shrews showed evidence of moderate rhinitis and one showed severe rhinitis. Tracheitis, characterized desquamated cilia and incrassate epithelium, was evident in one mild case and one moderate case in nine tree shrews inoculated with V0215 and three mild cases in nine tree shrews inoculated with Y12. Pneumonia was also evident as pulmonary interstitial thickening filled with inflammatory cells (Fig. 7). Five mild cases, one moderate case and one severe case of pneumonia were observed in seven tree shrews inoculated with V0215. Two mild, three moderate and two severe cases of pneumonia were observed in nine tree shrews inoculated with Y12. Overall, it appeared that Y12 caused more severe disease in tree shrews compared to V0215.

Histopathology of respiratory tissues obtained from ferrets revealed rhinitis and pneumonia in both V0215 and Y12 inoculated ferrets at 2, 4 and 6DPI. A mild tracheitis characterized by few desquamated cilia was only evident in one Y12 inoculated ferret at 2DPI (Fig. 8). A moderate rhinitis characterized by hyperemia and inflammatory cells in the submucosal layer of the nasal turbinate was observed in Y12 inoculated ferrets at 2 and 4DPI (Fig. 8d and j, respectively) and in a V0215 inoculated ferret at 2DPI (Fig. 8a). Severe rhinitis was also observed in a Y12 inoculated ferret at 6DPI (Fig. 8p) and V0215 inoculated ferrets at 4 and 6DPI (Fig. 8g and m, respectively). Interstitial pneumonia characterized by pulmonary interstitial thickening filled with inflammatory cells was observed in both V0215 and Y12 inoculated ferrets. Severe pneumonia was also observed in a V0215 inoculated ferret at 2DPI (Fig. 8c), although in inoculated ferrets examined at 6DPI, evidence of inflammation was mild (Fig. 8i and o). Moderate pneumonia was observed in Y12 inoculated ferrets at 2, 4 and 6DPI (Fig. 8f, l and r, respectively). Overall, there were no obvious differences between ferrets inoculated with V0215 or Y12.

After inoculation with Y12, interstitial pneumonia was observed in all but two mice (Fig. 9). Of those 15 mice inoculated with V0215, 9 mice showed severe pneumonia; five at 4DPI and four at 6DPI. Four mice showed pneumonia of moderate severity; three at 2DPI and one at 6DPI. Two mice showed pneumonia of mild severity at 2DPI. Of mice inoculated with Y12, 1 of 15 was killed at 5DPI. Three mice showed severe pneumonia, two at 4DPI and one at 6DPI. Five mice showed moderate pneumonia; two at 2DPI, two at 4DPI and one at 6DPI. Overall, V0215 appeared to cause a more severe disease in mice compared to Y12.

## Discussion

As one of the major causes of seasonal influenza, IBV seriously threatens human health. As vaccines and antiviral compounds are the main interventions against IBV, it is essential to have animal models that replicate human disease to use in their development. We previously inoculated mice with IBVs isolated from clinical samples and identified two IBVs capable of causing lethal infections in mice without adaptation; the Victoria-like virus B/Guangzhou/0215/2012 (V0215) and the Yamagata-like virus B/Guangzhou/12/2016 (Y12). In this current study, we used these viruses to compare IBV infection in tree shrews to ferrets and mice, to determine if tree shrews could be a model for future studies of IBVs.

Previously, we demonstrated that tree shrews present a useful alternative model for the study of subtype H1N1 IAV infection. Evidence of viral replication in the upper respiratory tract was observed, as were fever, nasopharyngeal secretions and inflammation. Additionally, the distribution of α2,3-linked and α2,6-linked sialic acids were similar to that found in the human respiratory tract. However, the effects of IBV in tree shrews were unknown. Our results showed that Yamagata and Victoria-lineage IBVs were capable of replication in the respiratory tract of tree shrews, ferrets and mice. Clinical signs and pathological changes were also evident in the respiratory tracts of these animals. Ferrets infected with influenza B virus may have mild respiratory and systemic symptoms, including fever, runny nose, sneezing, however, these nasal and systemic symptoms of tree shrews are not as obvious as ferrets, But in other aspects, tree shrews appeared to be more sensitive to IBV inoculation compared to ferrets, as weight loss was observed in tree shrews but not in ferrets. However, the overall severity of infection in these two animal models remained mild, which is comparable to what is observed in humans. In the course of this study, we observed that the main symptoms in mice infected with influenza virus include ruffled fur, arched back, shortness of breath and malaise. From the analogue comparison of symptoms after virus infection, tree shrews and ferrets were superior to mice as a model for human IBV infection. Effective replication of viruses in host is the main pathogenic mechanism. Previously, it was considered that unadapted influenza B virus could not be infected or replicated effectively in laboratory animals such as mice [16, 17], because influenza B virus exhibit a limited host range and lacked natural animal hosts [18, 19]. In this study, however, tree shrews, ferrets and mice were susceptible to two lineages of unadapted human influenza B (V0215 and Y12). The results showed that the respiratory tract of these animals infected with influenza B virus could replicate the virus effectively, which the peak of virus replication was 2 days after infection. The peak and period of respiratory viral shedding of tree shrews infected with influenza B virus were similar to those of ferrets, and similar to the respiratory viral shedding symptoms of human influenza diseases,such as viral shedding time of duration and peak time of viral shedding. [20, 21], whereas the viral shedding cycle of mice increased significantly, and there were still high viral titers in the lung tissues until 6DPI. We also found differences in susceptibility of three animals to above two influenza B viruses. There were no significant differences in titers between V0215 and Y12 inoculated ferrets, however, viral titers were significantly higher in tree shrews and mice inoculated with Y12 compared to V0215 at both 2 and 4DPI. This difference may be related to the different virulence and tissue tropism of V0215 and Y12, as well as host differences [22, 23]. Although there was no difference in viral titers between tree shrews and ferrets infected with Y12, viral titers were detected at 6DPI in tree shrews but not in ferrets. Histopathology of respiratory tissues obtained from tree shrews inoculated by IBVs revealed rhinitis and pneumonia in all animals. Because Y12 has longer viral shedding time and higher viral titer in tree shrews, it appeared that Y12 caused more severe disease in tree shrews compared to V0215 by pathological examination.

The antibody response of hosts infected with influenza virus is an important immune and epidemic prevention mechanism. Highly virulent influenza virus strains produce higher antibody titers, which are related to the severity of influenza infection [24, 25]. A survey of serum antibodies against influenza viruses in Pennsylvania showed that the titers of antibodies in most people infected with A/California/7/2009 (H1N1) and B/Brisbane/60/2008 (B/Victoria) ranged from 0 to 1:640 and 0–1:640, respectively. A few patients infected with H1N1 virus can reach 1:1280 in some months, some even greater than 1:2560 [26], Tree shrews and ferrets infected with IBV 0215 (B/Victoria) and 12 (B/Yamagata) can seroconvert, indicating that tree shrews have certain similarities in adaptive immunity with humans and ferrets, and could therefore be used in the development and research of new vaccines.

The immune response formed by cytokines and chemokines is the first line of defense against influenza virus infection and the key factor of the immune pathogenesis of influenza virus infection in human and animal models [27]. Moderate inflammation can inhibit the replication and transmission of influenza virus and prevent the development of disease, while excessive inflammation is closely related to clinical severe pathological injury [28]. Especially in some highly pathogenic avian influenza patients, excessive inflammation can promote the imbalance of immune function and the excessive release of a large number of inflammatory cytokines, termed a “cytokine storm”, leading to multiple organ dysfunction or acute respiratory distress syndrome [29]. In this study, we detected six cytokines in respiratory tract tissues of tree shrews, ferrets and mice. Elevated levels of cytokines were detected in the tissues isolated from the respiratory tract of tree shrews, ferrets and mice after infection with either V0215 or Y12 compared to the levels in the uninfected control, suggesting that infection with IBV can trigger innate immune responses in experimental animals including tree shrews. The expression of these cytokines correlated well with the pathological changes of respiratory tract tissues, as observed in similar reports using the ferret model [30, 31]. Similarly, many pro-inflammatory cytokines and chemokines such as IL-6, IL-8, IP-10, MIG, TNF-α and so on were significantly up-regulated in Influenza virus infection patients [32, 33]. In addition, IL-10 is an anti-inflammatory cytokine, which can strongly inhibit the synthesis of TNF-α, IL-1, IL-6, IL-8 and other inflammatory mediators. In this study, IL-10 increased significantly in tree shrews and mice tissues, suggesting that similar to human infection, tree shrews infected with influenza can also form a feedback regulation mechanism between pro-inflammatory and anti-inflammatory responses, maintaining the balance between inflammatory and anti-inflammatory mediators in the body [34].

Therefore, considering their small size, ease of handling and reduced cost of the tree shrews in comparison to ferrets, they present as a useful model for the study of IBV infection.

## Conclusion

Our data shows that two human IBVs, Y12 and V0215, which cause lethal infections in mice, can replicate and cause pathological changes in the respiratory tract of ferrets and tree shrews. Further, we observed that tree shrews display clinical signs such as fever, weight loss and nasal exudates following IBV inoculation that were either absent or milder in ferrets. In conjunction with their ease of handling, small size and cost effectiveness, the tree shrew model represents a viable animal model for the study of human IBVs.

## Additional file

Additional file 1: **Table S1.** The primers of ferret, tree shrew and mice. (DOCX 15 kb)

## Abbreviation

BSL: Biosafety Level
DPI: Days post inoculation
HI: Hemagglutination inhibition
IAVs: Influenza A viruses
IBVs: Influenza B viruses
IVC: Independent ventilation cage
MDCK: Madin–Darby canine kidney cell
TCID_50_: 50% Tissue culture infective dose
TPCK: L-(tosylamido-2-phenyl) ethyl chloromethyl ketone
V0215: Influenza virus B/Guangzhou/0215/2012
Y12: Influenza virus B/ Guangzhou/12/2016
