# Supplementary material for: The tree shrew is a promising model for the study of influenza B virus infection
Source: Virol J. 2019 Jun 7;16:77. doi: 10.1186/s12985-019-1171-3 (PMC6555921; doi:10.1186/s12985-019-1171-3)
Supplement: Supplementary file 1 — Table S1. The primers of ferret, tree shrew and mice. (DOCX 15 kb) [file 12985_2019_1171_MOESM1_ESM.docx]

**Supplement**

Table 1. The primers of ferret, tree shrew and mice

| species | gene | F/R | Sequence (5’ to 3’) |
| --- | --- | --- | --- |
| Ferret | GAPDH | F | CTGAACCCCAAAGCCAACCG |
|  |  | R | CCCCAGAGTCCATGACAATA |
|  | IL-6 | F | AAATGTGAAGACAGCAAGGAGG |
|  |  | R | GATTGAATTGAGACTGGAAGCA |
|  | CXCL8 | F | CACTCCACGCCTTTCCATC |
|  |  | R | CCAGTCTTCCTTGGGGTCC |
|  | IL-10 | F | TTGTTGACTGGGTCCTTGCT |
|  |  | R | ACGTGCTCCTTGACTTCTGG |
|  | CXCL10 | F | TTGAGGTGATTCCAGCAAGTCAATC |
|  |  | R | GACCTTTCCTTGCTAATTGCTTTCA |
|  | TNF-α | F | CGCTTCGCTGTCTCCTACCA |
|  |  | R | TCGATCCCCCTTCTCCAGTT |
|  | TGF-β | F | CTGCCCCTACATTTGGAGCCTG |
|  |  | R | GCTTGCGGCCCACGTAGTACAC |
| Tree shrew | GAPDH | F | TCGGAGTAAACGGATTTG |
|  |  | R | CCATGTAGTTCAGGTCAA |
|  | IL-6 | F | ACCAGAACCCACCTCCACAGATAAA |
|  |  | R | GGAAGGTTCAGATTGTTTTTTGCCAGT |
|  | CXCL8 | F | AAGACACATTCGACGCCTTTTCAC |
|  |  | R | ACCTTCTGCACCCACTTTTCCTTG |
|  | IL-10 | F | GTGAGAACAAGAGCAAGG |
|  |  | R | GGCTTTGTAGACACCTTTC |
|  | CXCL10 | F | GCTGTACGTGCATCACCATCAGTAA |
|  |  | R | CTGCTTTCAGTAGGTTCTTGATGGC |
|  | TNF-α | F | GCGTGCCAACACCCTCCTGA |
|  |  | R | CACAGGGGTGGAGGGGCAGC |
|  | TGF-β | F | AGCAGCACGTAGAACTGTACCAGAAA |
|  |  | R | AAAGCCCTCTATTCCCTCTCTGCG |
| Mice | GAPDH | F | ATCTGGCACCACACCTTCTACAA |
|  |  | R | TGATCTGGGTCATCTTTTCACGG |
|  | IL-6 | F | ACAAGAAAGACAAAGCCAGAGTCC |
|  |  | R | CTGTTAGGAGAGCATTGGAAATTG |
|  | CXCL8 | F | CCTGTGACACTCAAGAGCTACGA |
|  |  | R | GGGACTGCTATCACTTCCTTTCT |
|  | IL-10 | F | TTTGAATTCCCTGGGTGAGAA |
|  |  | R | GCTCCACTGCCTTGCTCTTATT |
|  | CXCL10 | F | TGAGGGCCATAGGGAAGCTTGAAAT |
|  |  | R | TCCGGATTCAGACATCTCTGCTCAT |
|  | TNF-α | F | CCAACGCCCTCCTGGC |
|  |  | R | GCAAATCGGCTGACGGTGT |
|  | TGF-β | F | TGTCTTTTGACGTCACTGGAGT |
|  |  | R | CGTGGAGTTTGTTATCTTTGCT |
